# Epoxyeicosatrienoic Acid and Prostanoid Crosstalk at the Receptor and Intracellular Signaling Levels to Maintain Vascular Tone

**DOI:** 10.3390/ijms23115939

**Published:** 2022-05-25

**Authors:** Pedro Felipe Malacarne, Justus Bezzenberger, Melina Lopez, Timothy Warwick, Niklas Müller, Ralf P. Brandes, Flávia Rezende

**Affiliations:** 1 Institute for Cardiovascular Physiology, Goethe-University, 60590 Frankfurt, Germany; malacarne@vrc.uni-frankfurt.de (P.F.M.); bezzenberger@vrc.uni-frankfurt.de (J.B.); mlopez@vrc.uni-frankfurt.de (M.L.); warwick@vrc.uni-frankfurt.de (T.W.); nmueller@vrc.uni-frankfurt.de (N.M.); brandes@vrc.uni-frankfurt.de (R.P.B.); 2 German Centre for Cardiovascular Research (DZHK), Partner Site Rhein-Main, 60590 Frankfurt, Germany

**Keywords:** epoxyeicosatrienoic acid, nitric oxide, thromboxane, prostaglandins, arachidonic acid

## Abstract

Epoxyeicosatrienoic acids (EETs) are signaling lipids produced by the cytochrome P450-(CYP450)-mediated epoxygenation of arachidonic acid. EETs have numerous biological effects on the vascular system, but aspects including their species specificity make their effects on vascular tone controversial. CYP450 enzymes require the 450-reductase (POR) for their activity. We set out to determine the contribution of endothelial CYP450 to murine vascular function using isolated aortic ring preparations from tamoxifen-inducible endothelial cell-specific POR knockout mice (ecPOR^−/−^). Constrictor responses to phenylephrine were similar between control (CTR) and ecPOR^−/−^ mice. Contrastingly, sensitivity to the thromboxane receptor agonist U46619 and prostaglandin E2 (PGE2) was increased following the deletion of POR. Ex vivo incubation with a non-hydrolyzable EET (14,15-EE-8(Z)-E, EEZE) reversed the increased sensitivity to U46619 to the levels of CTR. EETs had no effect on vascular tone in phenylephrine-preconstricted vessels, but dilated vessels contracted with U46619 or PGE2. As U46619 acts through RhoA-dependent kinase, this system was analyzed. The deletion of POR affected the expression of genes in this pathway and the inhibition of Rho-GTPase with SAR407899 decreased sensitivity to U46619. These data suggest that EET and prostanoid crosstalk at the receptor level and that lack of EET production sensitizes vessels to vasoconstriction via the induction of the Rho kinase system.

## 1. Introduction

**Figure 1:**
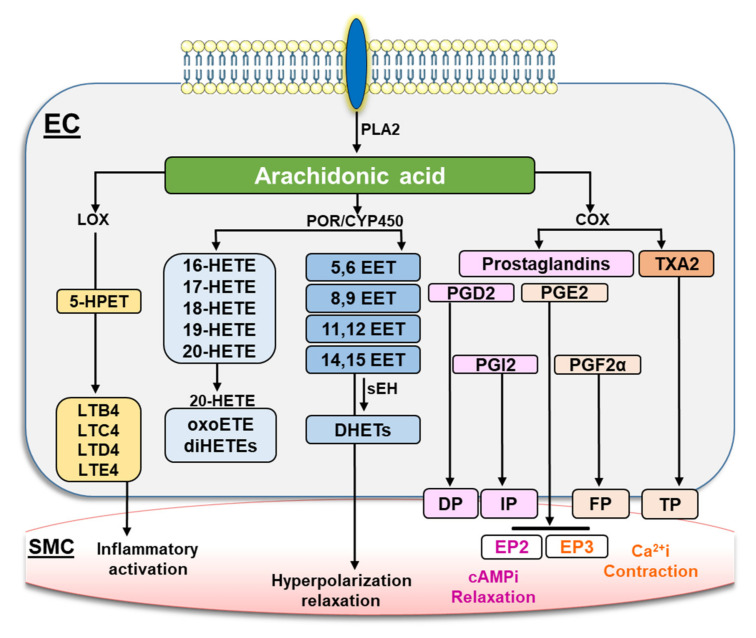
**Metabolic pathways of arachidonic acid.** Phospholipase A2 (PLA2) hydrolyzes arachidonic acid (AA) from membrane-bound phospholipids. The cyclooxygenase enzymes (COX) metabolize AA into different prostanoids such as prostaglandin D2 (PGD2), prostacyclin (PGI2), prostaglandin E2 (PGE2), prostaglandin F2α (PGF2α) and thromboxane A2 (TXA2). These prostanoids act on their respective preferential receptor, DP, IP, EP, FP and TP, leading to control of vessel tone. AA can also be metabolized through the cytochrome P450 reductase (POR)/CYP450 system leading to production of 5,6-; 8,9-; 11,12-; and 14,15-epoxyeicosatrienoic acids (EETs) as well as several biologically active hydroxyeicosatetraenoic acids (HETEs). The EETs can be further catabolized to the less-active dihydroxyeicosatrienoic acid (DHET) through the soluble epoxide hydrolase enzyme (sEH). The lipoxygenases (LOX) metabolize AA into leukotrienes (LTB4, LTC4, LTD4 and LTE4) through reactions that can also involve the formation of an intermediate: 5(S)-Hydroperoxyeicosatetraenoic acid (5-HPET).

Oxidation products of arachidonic acid (AA) are important signaling lipids with numerous effects on the cardiovascular system [1,2]. AA is released from membrane phospholipids by phospholipase A2, either in response to its phosphorylation or following increases in intracellular Ca^+2^. Hydrolyzed AA is further metabolized by lipoxygenase (LOX), cyclooxygenase (COX) or cytochrome P450 (CYP450) enzyme systems. The resulting lipids are then re-esterified and stored in cellular membranes or act as signaling molecules in an autocrine or paracrine fashion (Figure 1) [1,3].

COX enzymes metabolize AA to endoperoxides, which are, depending on local enzyme abundance, converted to various prostanoids [4]. Prostanoids may act as both vasodilators (prostaglandin (PG) D2 and PGI2) and vasoconstrictors (PGE2, PGF2α and thromboxane (TXA2)). The direct effects of PG on the tone of smooth muscle cells (SMC) are mediated by the family of endoperoxide (EP)–G-protein coupled receptors [1,3]. Prostanoids are suggested to crosstalk at the receptor level. For example, TXA2 preferentially binds to thromboxane receptors (TP); however, it can synergize with PGE2 by acting on the EP3 receptor and antagonize the actions of PGI2 [5]. At the receptor level, TP forms heterodimeric complexes with prostacyclin receptors that enhance G_S_ signaling [6]. The activation of TP initiates G_q_ and G_12/13_ signaling, leading to inositol 1,4,5-triphosphate (IP_3_) synthesis and the activation of RhoA and RhoA-dependent kinase (Rock1/2). The latter elicits SMC contraction by reducing myosin light chain phosphatase (MLCP) activity [7,8].

The CYP450 hydroxylase system converts AA to 20-hydroxyeicosatetraenoic acid (20-HETE), whereas the CYP450-epoxygenases insert an epoxide at any of the four double bonds of AA, producing 5,6-; 8,9-; 11,12-; and 14,15-EET. In human, bovine and porcine vasculature, EETs elicit endothelium-derived hyperpolarization, which leads to vasodilation via the closure of L-type calcium channels [9].

EETs are further catabolized to dihydroxyeicosatrienoic acids (DHET) regioisomers by the enzyme sEH (soluble epoxide hydrolase). Due to the lack of specific CYP450 inhibitors and redundancy within the CYP450 system, the vascular effects of EETs have predominantly been studied using sEH loss of function models [10,11,12] or by the overexpression of CYP2C8 and CYP2C2 [13]. We have recently generated an alternative loss of function approach to study vascular EETs: an endothelial-cell-specific, tamoxifen-inducible cytochrome P450 reductase (POR) knockout mouse (ecPOR^−/−^). POR transfers electrons from NADPH to microsomal CYP450 enzymes. In the absence of POR, the microsomal CYP450 system is therefore inactive. ecPOR^−/−^ mice exhibited endothelial dysfunction, lower aortic and pulmonary EET levels and increased vascular prostanoid (thromboxane and PDG2) levels. The deletion of POR potentiated the hypertensive effect of angiotensin II. As the inhibition of COX blocked this effect, the loss of endothelial POR/CYP450 appears to induce a functional shunt of AA towards the vasoconstrictor COX pathway [14].

In this study, we set out to study the mechanisms of the crosstalk between EETs and the prostanoid system using isolated murine aortic ring preparations. We report that EETs act as competitive agonists of prostanoid receptors. Moreover, crosstalk between EETs and prostanoids takes place via Rho kinase signaling, and these mechanisms contribute to their effect on vascular tone.

## 2. Results

### 2.1. Knockout of Endothelial POR Increases Vessel Constriction to the Thromboxane Receptor Agonist U46619 or PGE2

**Figure 2:**
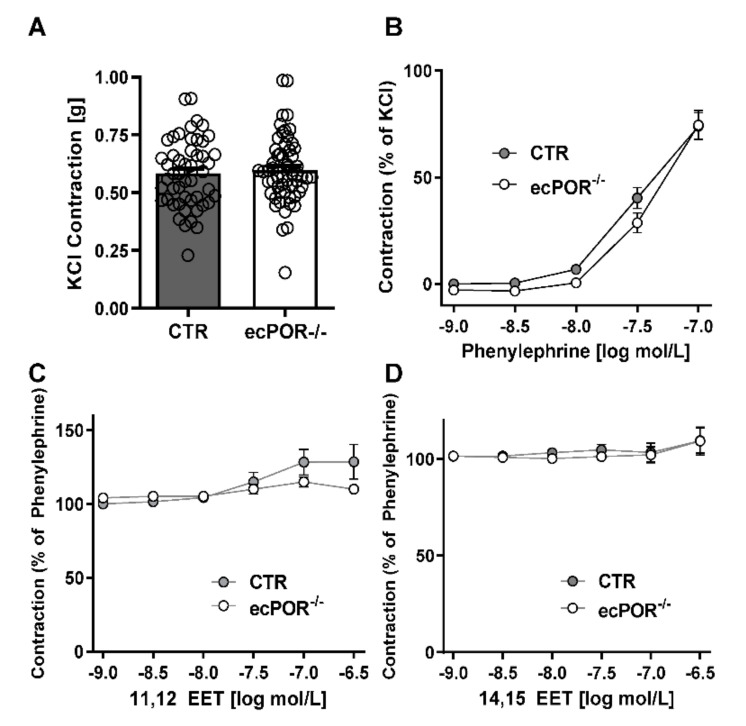
Epoxyeicosatrienoic acids do not modulate phenylephrine−induced vasoconstriction. (**A**) Constrictor response to KCl (80 mM) in aortic rings from mice of the genotype indicated. (**B**) Concentration curve for contraction to phenylephrine. (**C**) Concentration–relaxation curves to 11, 12-EET and (**D**) 14, 15-EET using aortic rings of CTR and ecPOR^−/−^ mice. n ≥ 12 aortic rings per condition. Two-way ANOVA with Bonferroni post-test.

To analyze the impact of the POR-dependent inactivation of CYP450 on vascular tone, we performed organ chamber experiments using aortic rings isolated from control (CTR) and ecPOR^−/−^ mice. Aortic rings from ecPOR^−/−^ mice showed no differences from CTR rings in their contractile response to KCl (80 mM, Figure 2A) or phenylephrine (Figure 2B). Neither 11,12-EET nor 14,15-EET induced relaxation in phenylephrine-preconstricted rings of either genotype (Figure 2C,D). These results are in line with previous findings stating that EETs do not relax mouse vessels that are preconstricted with phenylephrine [15,16]. These data suggest that in the mouse aorta, the effects of EETs on the vessel are not a consequence of a direct vasodilation response. In addition, the actions of EETs do not interfere with the signaling pathways of α1 adrenergic receptors or depolarization-induced constriction.

**Figure 3:**
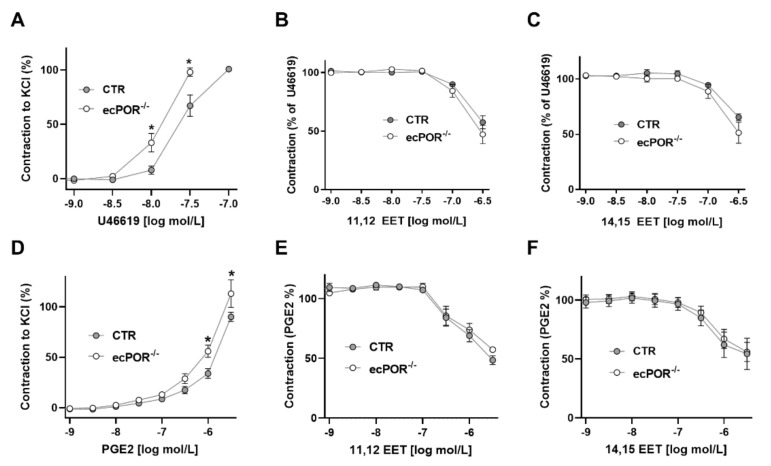
Knockout of POR in the endothelium leads to increased constriction of the thromboxane receptor agonist U46619 and PGE2 in aorta. Vasoconstriction of aortic vessels from CTR and ecPOR^−/−^ mice in response to U46619 (**A**) and PGE2 (**D**) * *p* < 0.05, Two-way ANOVA with Bonferroni post-test. n > 11 aortic rings. (**B**,**E**) Vasodilation of aortic vessels from CTR and ecPOR^−/−^ mice following 11,12-EET treatment after preconstriction with U46619 (**B**) and PGE2 (**E**). Two-way ANOVA with Bonferroni post-test. n ≥ 7 aortic rings. Vasodilation of aortic vessels from CTR and ecPOR^−/−^ mice in response to 14,15-EET after preconstriction with U46619 (**C**) and PGE2 (**F**). Two-way ANOVA with Bonferroni post-test (**C**) n = 4 and (**D**) n = 5 aortic rings.

As we had previously observed that the endothelial-specific deletion of POR increases aortic levels of thromboxane and PGD2, we next examined the constrictive response of the aorta to U46619. Aortic vessels from ecPOR^−/−^ mice showed a significantly higher sensitivity to the constrictor U46619 (EC50: approx. −7.8 log mol/L) as compared to vessels from CTR mice (EC50: −7.6 log mol/L) (Figure 3A). This suggests that the TP receptor is primed or the downstream signaling cascade is sensitized or induced in ecPOR^−/−^ mice. Interestingly, after U46619-mediated contraction, high concentrations of EETs were able to relax the vessels. This may suggest that EETs either compete with U46619 on the TP receptor or interfere with TP receptor signaling (Figure 3B,C). To differentiate between these two modes of action, the response of vessels to a second prostanoid constrictor, PGE2, was studied. PGE2 acts through a different receptor but via an analogous signaling pathway. Similarly to the thromboxane agonist, vessel constriction in response to PGE2 was sensitized by the deletion of POR (Figure 3D). As in the case of U46619, EETs also induced the relaxation of PGE2-preconstricted vessels (Figure 3E,F).

**Figure 4:**
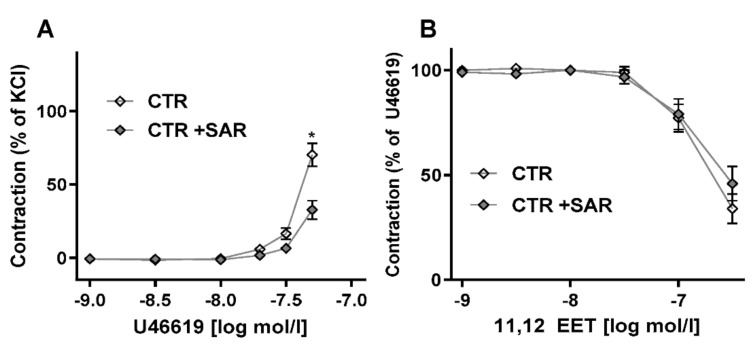
**Effect of the Rho-kinase inhibitor SAR407899 (SAR) on U46619 contraction and EET-induced relaxation.** Organ chamber experiments using aortic rings. (**A**): Constriction after U46619 stimulation with or without pre-incubation with SAR407899. (**B**): 11,12-EET-induced relaxation in U46619 preconstricted vessels incubated (+SAR) with or without SAR407899 * *p* < 0.05, Two-way ANOVA with Bonferroni post-test. n > 7.

In comparison to phenylephrine and KCl, the constriction induced by U46619 is largely mediated by RhoA-dependent kinase activation and light chain phosphatase inhibition [17]. Given that EETs have been linked to the RhoA-system, the effect of RhoA-dependent kinase inhibition on U46619-induced vasoconstriction and 11,12-EET-induced vasodilation were studied (Figure 4). As expected, RhoA-dependent kinase inhibition attenuated U46619-induced vasoconstriction, confirming the involvement of calcium sensitization in this response. Vasodilation in response to EETs remained unaffected. These data suggest that EETs act upstream of Rho A-dependent kinase. This could imply EETs act via direct ligand-dependent antagonism at the receptor level or by interference with the G-protein involved.

### 2.2. Restoring EET Pools in Aortic Rings of ecPOR^−/−^ Mice Normalizes Contraction to U46619

**Figure 5:**
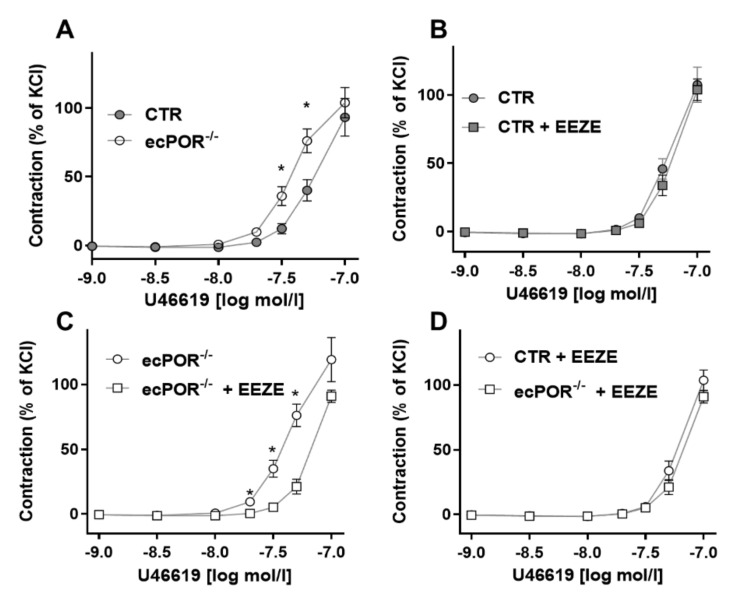
**14,15-EE-8(Z)-E incubation reverses the increased constriction to U46619 in the ecPOR^−/−^ mice.** Constriction of aorta from CTR and ecPOR^−/−^ mice in response to U46619 with or without pre-incubation with 14,15 EE-8(Z)-E (24 h, 10 µmol/L) as indicated. * *p* < 0.05, Two-way ANOVA with Bonferroni post-test. n ≥ 14 aortic rings.

To examine more chronic effects of EETs on the signaling cascade leading to TB-receptor-mediated constriction, aortic rings were kept in organ culture for 24 h in the presence or absence of the stable 14,15-EET analogue 14,15-EE-8(Z)-E (EEZE, 24 h, 10 µmol/L). EEZE-incubation had no effect on the constrictor response of CTR vessels but de-sensitized ecPOR^−/−^ vessels’ responses to U46619. After EEZE treatment, vasoconstriction in response to U46619 treatment was identical between vessels from both mouse strains (Figure 5). These results suggest that prolonged EET exposure impacts the signaling pathway of TP receptors.

### 2.3. Knockout of POR in Endothelial Cells (HUVEC) Increases Expression of Genes Related to Rho Signaling

**Figure 6:**
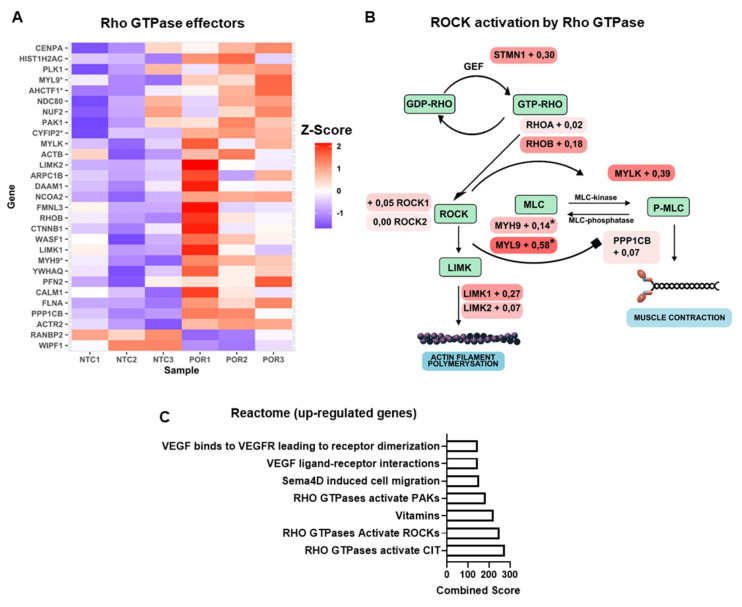
**Effect of deletion of POR in HUVEC in the Rho-kinase system.** (**A**) Heatmap of genes (*p* < 0.05) pertaining to the Rho GTPase effectors (Reactome) from RNA sequencing of NTC (non-targeting control) and POR^−/−^ CRISPR/Cas HUVEC (* FDR < 0.05). (**B**) Rho GTPase ROCK activation pathway with log2fold change values (POR^−/−^/NTC) for the genes of the associated pathway (* FDR < 0.05). (**C**) Pathway analysis using the Reactome-2016 dataset for differentially expressed genes from the HUVEC RNA sequencing. Bars represent combined scores from top seven analyzed pathways using *Enrichr*. FDR: false discovery rate.

To profile gene expression changes linked to lower EET production in endothelial cells, RNA-seq was performed in HUVEC after CRISPR/cas9-mediated deletion of POR (POR^−/−^) and HUVEC treated with non-targeting control sgRNA (NTC). Interestingly, gene set enrichment analysis of differentially expressed genes returned the term “RHO GTPases Activate ROCKs” as one of the most significantly regulated gene sets (Figure 6). This suggests that a lack of EETs may sensitize the Rho GTP pathway in general. These data should, however, be handled with caution, considering that vasoconstriction occurs in smooth muscle cells and EETs may have divergent functions in endothelial cells. Moreover, HUVEC are known to rapidly dedifferentiate with respect to the CYP450 system; hence, the data may not reflect the in vivo situation.

### 2.4. Addition of EET to Aortic Tissue Increases Expression of Rho Kinases

**Figure 7:**
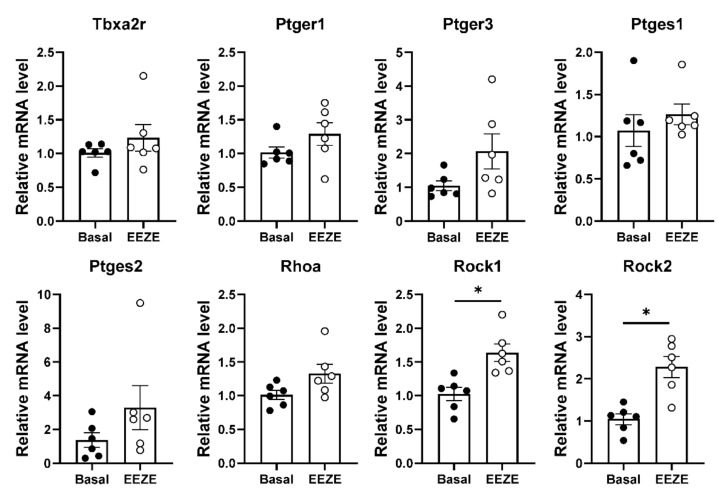
RT-qPCR for selected genes from aortic tissue pre-incubated with or without 14,15-EE-8(Z)-E. * *p* < 0.05, Mann–Whitney test. n = 6 mice.

To address the aforementioned limitations, we incubated wild-type (WT) aortic rings with EEZE and subsequently determined gene expression for selected genes by RT-qPCR. Interestingly, of the selected genes, only *Rock1* and *Rock2* displayed increased expression in response to EEZE (Figure 7). These data may reflect the fact that chronic exposure to EETs limits the activity of the Rho kinase system [18], and therefore, the induction of *Rock1* and *Rock2* expression represents a compensatory mechanism.

## 3. Discussion

EETs were first discovered almost forty years ago [19] and were described as potent vasodilators originating from the metabolism of AA by CYP450 enzymes. Their mode of dilation was first attributed to large-conductance Ca^2+^-activated K^+^ channels and/or transient receptor potential cation channel subfamily V, member (TRPV4) [20,21]. However, the vessel dilation induced by EETs in experiments using knockout mice for these channels revealed no differences as compared to wild-type mice [15], suggesting alternative receptors exist for EETs. The fact that EETs were only able to induce dilation of vessels preconstricted with the TP receptor agonist U46619 suggests that EETs act through TP receptors as competitive antagonists [15,16]. Competition assays with radioactive H^3^-14,15-EET and the use of specific inhibitors of the cAMP/PKA-dependent pathway further supported the notion that EETs bind to GPCR (guanine nucleotide-binding protein-coupled receptors) [22,23]. Among tested GPCRs, the TP receptor showed the highest Ki (6.1 µM) [15]. Additionally, isometric tension studies using rat mesenteric arteries showed that vasodilation in response to 14,15-EET was inhibited by AH6809, an EP2 receptor antagonist [24]. Thus, EETs—particularly 14,15-EET—are able to cross-activate prostanoid receptors. In the present study, we utilized a novel model to study the binding of EETs to TP and EP receptors: an endothelial cell-specific POR knockout mouse that abolishes the production of EETs [14]. In the absence of a functional CYP450 system, there was a functional shunt towards the COX pathway. Vascular TXA2 and PGE2 were significantly increased in aortic tissue of ecPOR^−/−^ mice compared to control mice. Importantly, increased prostanoid levels lead to hypertension (AngII infusion model). Hypertension observed in ecPOR^−/−^ mice was rescued by treatment with the non-selective COX inhibitor Naproxen [14]. We first hypothesized that a lack of EETs in conjunction with increased production of TXA2 and PGE2 could enhance the priming of TP and EP2, thereby facilitating vasoconstriction in ecPOR^−/−^ mice. The aortic constriction in response to phenylephrine was unchanged between vessels originating from CTR and ecPOR^−/−^ mice, and further addition of EETs could not induce vessel relaxation. These findings are in line with previous reports and confirm that EETs do not affect the activation of α1-adreno receptor signaling. In contrast, EET treatment induced relaxation of vessels preconstricted with U46619 and PGE2, further supporting the hypothesis that EETs act as competitive antagonists of prostanoid receptors. Importantly, aortae from ecPOR^−/−^ constrict significantly more in response to U46619 and PGE2 than those from control mice, suggesting that higher levels of TXA2 and PGE2 could prime their respective receptors, facilitating the constrictive response to their agonists. Our studies were focused on 11,12-EET and 14,15-EET, as these are the predominant isomers that exhibit the most potent effects in murine vascular tissue [25], although similar effects can be expected for 5,6- and 8,9-EETs. Incubation of vessels from ecPOR^−/−^ mice with EEZE normalized the increased constriction in response to U46619, suggesting that higher EET availability might result in higher occupancy of the TP receptor. As these results pointed to crosstalk at the signaling level, we further investigated how a lack of EETs affects intracellular downstream signaling triggered by prostanoids. Expression changes in genes annotated to Rho signaling could be observed in human endothelial cells lacking EETs following the genomic deletion of POR. Similarly, increased Rho signaling could be observed in aortic segments from wild-type mice that were exposed to an excess of EETs (incubated with EEZE), showcased by significantly increased expression of *Rock1* and *Rock2*. These results suggest that any changes in EET pools can be compensated for at the level of intracellular signaling but seemingly not via expression changes of the prostanoid receptors. Functionally, the inhibition of Rho signaling with SAR407899 increased the threshold for constriction in response to U46619. Altogether, we observe a crosstalk between EETs and prostanoids at the receptor and intracellular signaling levels by using aortae from the ecPOR^−/−^ mouse model and wild-type mice supplemented with EEZE or treated with SAR407899 to inhibit Rho signaling.

## 4. Materials and Methods

### 4.1. Chemicals

All chemicals, if not otherwise specified, were purchased from Sigma Aldrich (St. Louis, MO, USA). 11,12-EET (#50511); 14,15-EET (#50651); 14,15-EE-8(Z)-E (#10010486); PGE2 (#14010); and U46619 (#16450) were from Cayman Chemical (MI, USA).

### 4.2. Animal Procedure

Wild-type mice (C57Bl6J) were purchased from Charles River (Sulzfeld, Germany). Endothelial cell-specific, tamoxifen-inducible POR knockout mice (ecPOR^−/−^) were generated as previously described [14]. Briefly, Por^flox/flox^ mice (cytochrome P450 reductase, Por^tm1^Ding, kindly provided by Xinxin Ding, University of Arizona, USA) were crossed with Cdh5-CreERT2 (Tg(Cdh5-CreERT2)^1^Rha) mice (kindly provided by Ralf Adams, Münster, Germany). POR deletion was induced by providing tamoxifen in the diet (400 mg/kg, 10 days) when animals were at least 8 weeks old. A tamoxifen-free “wash out” period of at least 14 days after tamoxifen feeding was adhered to. Control animals (CTR) are defined as Por^flox/flox^-Cdh5-CreERT2^0/0^ littermates (i.e., no Cre expression) and were also fed with tamoxifen. ecPOR^−/−^ denotes Por^flox/flox^-Cdh5-CreERT2^TG/0^ after tamoxifen treatment. All animals had free access to chow and water in a specified pathogen-free facility with a 12 h day/12 h night cycle, and all animal experiments were performed in accordance with the German animal protection law and were carried out after approval by the local authorities (Regierungspräsidium Darmstadt, under the approval FU1188). Every mouse received an identification number for each experiment, and the experimenter was blind to the genotype. Animal group sizes differ due to the number of littermates. Control and knockout animals were studied in a paired fashion per experiment, and the order was alternated daily.

### 4.3. Vascular Reactivity Measurements

Organ chamber experiments were performed using isolated aortic vessel segments (1 mm rings) in a carbogen-aerated Krebs–Henseleit buffer (37 °C, pH 7.4). Cumulative concentration–response curves were generated to phenylephrine (0.03–0.3 µmol/L), U46619 (0.03–0.3 µmol/L) and PGE2 (0.03–0.3 µmol/L) to obtain a preconstriction level of 80% of the initial KCl (80 mmol/L) constriction. Once a stable contraction was achieved, 11,12-EET and 14,15-EET (10 nmol/L–10 µmol/L) were added cumulatively to generate concentration–relaxation curves. Data were acquired and analyzed with the LabChart V8 software from ADInstruments (Oxford, United Kingdom).

### 4.4. EET Supplementation

Aortic vessel segments (1 mm rings) were incubated in the presence or absence of 14,15-EE-8(Z)-E (10 µmol/L) in endothelial basal medium (EBM, Pelo Biotech, Planegg, Germany) (24 h, 37 °C). After incubation with EEZE, aortic rings were either used for organ chamber experiments or snap-frozen for RNA isolation and RT-qPCR.

### 4.5. RNA Isolation and RT-qPCR

Total RNA was extracted with RNeasy Mini Kit (Qiagen, #74106) according to the manufacturer’s protocol. Briefly, frozen thoracic aortic tissue (~ 5 mm) was homogenized (TissueLyser LT, Qiagen) in 350 µL RLT buffer containing β-mercaptoethanol (10%). cDNA synthesis was carried out using SuperScript III reverse transcriptase (Thermo Fisher Scientific, # 12574026, Massachusetts, USA) and a combination of oligo(dT)23 and random hexamer primers (Sigma). Quantitative Real-time PCR was performed using iTaq Universal SYBR Green Supermix with ROX as reference dye (Bio-Rad, #1725121) in an AriaMX cycler (Agilent Technologies). Mouse target genes were normalized to eukaryotic elongation factor 2 (EEF2). Relative expression was calculated using the ∆∆Ct method with AriaMX qPCR software (Agilent). Primers used for amplification are listed in Table 1.

### 4.6. CRISPR/Cas9 for Cytochrome P450 Reductase (POR)

CRISPR/Cas9 knockout HUVEC (Human umbilical vein endothelial cells) for POR were generated with a lentiviral system. Guide RNAs for POR or non-targeting control (NTC) guides were designed using the publicly available CRISPR algorithm (www.benchling.com (accessed on 1 October 2016).) as previously described [26]. Oligonucleotides were cloned into the vector using the BsmBI restriction site. After cloning, the gRNA-containing vectors were purified and sequenced. Pseudotyped lentivirus was produced by transfection using PEI (polyethyleneimine, Sigma Aldrich) of LentiX cells (Takara, Japan) with LCV2 plasmid together with lentiviral packaging plasmid (Addgene #12260) and VSV-G (Addgene #12259). Viral supernatants were collected, filtered and snap-frozen on the third day after transfection. HUVEC (at passage 1) were transduced with viral particles for 24 h using polybrene (Sigma Aldrich, 8 µg/mL) and then selected with puromycin (2 µg/mL) for 4 days. Knockout efficiency was controlled by Western blotting.

### 4.7. RNA Seq from HUVEC

Total RNA from HUVEC was isolated with the RNA Mini Kit from Bio&SELL (Nuremberg, Germany) combined with on-column DNase digestion (DNase-Free DNase Set, Qiagen) to avoid contamination by genomic DNA. RNA and library preparation integrity were verified with LabChip Gx Touch 24 (Perkin Elmer, Massachusetts, EUA). A total of 2 µg of total RNA was used as input VAHTS Stranded mRNA-seq Library preparation following the manufacturer’s protocol (Vazyme, Nanjing, China). Sequencing was performed on the NextSeq2000 instrument (Illumina, Cambridge, United Kingdom) using P2 flow cell with v3 chemistry, resulting in an average of 45 M reads per library with 1 × 72 bp paired-read setup. The sequencing reads for all samples were quantified against the hg38 transcriptome (obtained from Ensembl (https://doi.org/10.1093/nar/gkz890 (accessed on 19 October 2021)) using Salmon (1.5.2) (https://doi.org/10.1038/nmeth.4197 (accessed on 19 October 2021)). Reads not aligned to the transcriptome were discarded at this point. Differential gene expression analysis was performed using DESeq2 (1.32.0) (https://doi.org/10.1186/s13059-014-0550-8 (accessed on 19 October 2021)) in R (4.1.1) (R Core Team (2021). R: A language and environment for statistical computing. R Foundation for Statistical Computing, Vienna, Austria). Raw transcript counts were summed per gene and used in the standard DESeq2 differential gene expression analysis workflow, using a negative binomial test over gene counts in each of the combinations of conditions. Batch information was also included in the contrast formula.

### 4.8. Statistics

Unless otherwise indicated, data are given as means ± standard error of mean (SEM). Calculations were performed with Prism 8.0 or R (package ggplot2). In case of multiple testing, Bonferroni or Tukey correction was applied. For multiple group comparisons, ANOVA followed by post hoc testing was performed. Individual statistics of unpaired samples were performed by t-test and if not normally distributed, then by Mann–Whitney test. Measurements of vascular reactivity were analyzed with Two-way ANOVA with Bonferroni correction for repeated measurements. A *p*-value of <0.05 was considered as significant. n indicates the number of individual experiments, animals or aortic rings.

## 5. Conclusions

In conclusion, we show that EETs antagonize the action of vasoconstrictor lipids on the TXA2 and EP3 receptor. We also demonstrate that aortae of endothelial-cell-specific POR mice have a lower constriction threshold for the thromboxane receptor agonist U46619 and PGE2, which could be reversed by EEZE supplementation as is likely mediated by the Rho signaling cascade.

## Figures and Tables

**Table 1:** Primer list for RT-qPCR.

Name	Forward Primer (5′-3′)	Reverse Primer (5′-3′)
*Eef2*	GACATCACCAAGGGTGTGCAG	GCGGTCAGCACACTGGCATA
*Tbxa2r*	TGGTTCAGCTCGTGGGCATCAT	ACACGCAGGTAGATGAGCAGCT
*Ptger1*	TCATGGTGGTGTCGTGCATCTG	GTCCAGGATCTGGTTCCACGAT
*Ptger3*	GCTTCGCTGAACCAGATCTTGG	CAGGTACTGCAATGAAAGTCCAC
*Ptges1*	GAATGCCACCTTCATCCGAGAAG	GCTCACATTGGAGAAGGACTCC
*Ptges2*	GCGACATACTCAAGCAGGAGCA	AGTGGTAACCGCTCAGGTGTTG
*Rock1*	CACGCCTAACTGACAAGCACCA	CAGGTCAACATCTAGCATGGAAC
*Rock2*	GTGACCTCAAACAGTCTCAGCAG	GACAACGCTTCTGAGTTTCCTGC
*Rhoa*	CTTCAGCAAGGACCAGTTCCCA	GGCGGTCATAATCTTCCTGTCC

## Data Availability

Not applicable.
